# Supplementary material for: Coronary plaque composition influences biomechanical stress and predicts plaque rupture in a morpho-mechanic OCT analysis
Source: eLife. 2021 May 11;10:e64020. doi: 10.7554/eLife.64020 (PMC8112861; doi:10.7554/eLife.64020)
Supplement: Supplementary file 1. — Maximum and minimum element size, respectively, limits how big and how small each mesh element can be. Maximum element growth rate limits the size difference of two adjacent mesh elements. Curvature factor limits how big a mesh element can be along a curved boundary. Resolution of narrow regions controls the number of layers of mesh elements in narrow regions. [file elife-64020-supp1.docx]

| **Simulation** | **Maximum element size (mm)** | **Minimum element size (mm)** | **Maximum element growth rate** | **Curvature factor** | **Resolution of narrow regions** | **Number of elements** |
| --- | --- | --- | --- | --- | --- | --- |
| #1 | 0,55 | 0,04 | 1,4 | 0,4 | 0,7 | 255220 |
| #2 | 0,8 | 0,05 | 1,45 | 0,5 | 0,6 | 209568 |
| #3 | 0,55 | 0,04 | 1,4 | 0,4 | 0,7 | 45697 |
| #4 | 0,576 | 0,419 | 1,4 | 0,4 | 0,7 | 46356 |
| #5 | 0,356 | 0,0152 | 1,35 | 0,3 | 0,85 | 168106 |
| #6 | 0,8 | 0,055 | 1,45 | 0,5 | 0,6 | 345125 |
| #7 | 0,55 | 0,04 | 1,4 | 0,4 | 0,7 | 324940 |
| #8 | 0,8 | 0,1 | 1,45 | 0,5 | 0,6 | 208811 |
| #9 | 0,55 | 0,04 | 1,4 | 0,4 | 0,7 | 269874 |
| #10 | 0,55 | 0,04 | 1,4 | 0,4 | 0,7 | 411783 |
| #11 | 0,35 | 0,015 | 1,35 | 0,3 | 0,85 | 480374 |
| #12 | 0,35 | 0,015 | 1,35 | 0,3 | 0,85 | 408255 |
| #13 | 0,55 | 0,04 | 1,4 | 0,4 | 0,7 | 389769 |
| #14 | 0,55 | 0,04 | 1,4 | 0,4 | 0,7 | 402969 |
| #15 | 0,55 | 0,04 | 1,4 | 0,4 | 0,7 | 245099 |
| #16 | 0,8 | 0,05 | 1,45 | 0,5 | 0,6 | 408055 |
| #17 | 0,557 | 0,0405 | 1,4 | 0,4 | 0,7 | 269942 |
| #18 | 0,55 | 0,04 | 1,4 | 0,4 | 0,7 | 369736 |
| #19 | 0,55 | 0,04 | 1,4 | 0,4 | 0,7 | 482735 |
| #20 | 0,55 | 0,04 | 1,4 | 0,4 | 0,7 | 524533 |

**Supplementary Tables**

**Suppl. Table 1. Characteristics of the mesh.** Maximum and minimum element size respectively limits how big and how small each mesh element can be. Maximum element growth rate limits the size difference of two adjacent mesh elements. Curvature factor limits how big a mesh element can be along a curved boundary. Resolution of narrow regions controls the number of layers of mesh elements in narrow regions.
